# Sex differences in the effects of 10 Hz and 40 Hz transcranial alternating current stimulation on spatial cognition in mice

**DOI:** 10.1186/s13293-025-00778-5

**Published:** 2025-11-06

**Authors:** Yunbin Zhang, Ping Ren, Zhuangfei Chen, Yu Fu

**Affiliations:** 1 Medical School, Kunming University of Science & Technology, #727 Jing Ming Nan Road, Chenggong County, Kunming, 650500 Yunnan China; 2 Department of Geriatric Psychiatry, Shenzhen Mental Health Center/Shenzhen Kangning Hospital, Shenzhen, 518020 Guangdong China

**Keywords:** TACS, Sex differences, Spatial cognition, Locomotion, Anxiety, Mice

## Abstract

**Background:**

Sex differences are crucial to understanding neuropsychiatric disorders, yet they are often overlooked in the development of therapies. Transcranial alternating current stimulation (tACS) shows promise for cognitive enhancement, but its sex-specific effects are largely unknown.

**Methods:**

In this study, the effects of 10 Hz and 40 Hz tACS on spatial cognition were examined in male and female mice using three tests: the Y-maze to evaluate spatial recognition memory, the Barnes maze to evaluate spatial learning and memory related to punishment, and the reversal Barnes maze to evaluate reversal learning. General behaviors, such as anxiety, exploration, and locomotion, were evaluated using the elevated plus maze and open field tests.

**Results:**

The results showed that 40 Hz tACS improved spatial recognition memory in males, while 10 Hz and 40 Hz tACS enhanced spatial learning in females. Males learned faster, while females performed better initially in the spatial learning process. In addition, no significant effects of tACS were observed in reversal learning, spatial memory, anxiety, or exploration. Interestingly, males exhibited reduced locomotion compared to females across tasks, and tACS potentially exacerbated this difference.

**Conclusions:**

This animal study suggests that tACS may influence spatial cognition differently in males and females. Our findings highlight the importance of considering the interaction between sex and stimulation frequency when optimizing tACS intervention parameters.

## Abstract (plain language summary)

Transcranial alternating current stimulation (tACS) is a noninvasive brain stimulation technique that shows promise in improving cognitive function. In this study, we tested how different frequencies of tACS (10 Hz “alpha” and 40 Hz “gamma”) affect spatial cognition in male and female mice using tasks like the Y-maze, Barnes maze, and reversal Barnes maze, along with tests for general behaviors such as anxiety, exploration, and locomotion. The results showed clear sex and frequency differences: 40 Hz tACS improved spatial recognition memory in male mice, while 10 Hz and 40 Hz tACS enhanced spatial learning in female mice. Males learned faster, and females performed better initially in spatial learning. Importantly, tACS did not alter anxiety, exploration, or complex learning tasks. Notably, males moved less than females across all tests, and tACS seemed to increase this difference. These findings show that tACS affects male and female brains differently: males may need 40 Hz for memory enhancement, while females respond to both frequencies for learning. This highlights the need to customize brain stimulation treatments by sex for cognitive disorders.

## Highlights

- 40 Hz tACS improved male spatial recognition memory, while 10/40 Hz improved female spatial learning.
- tACS barely affected reversal learning, punishment-related memory, or anxiety.
- tACS exacerbated the difference in locomotion between males and females, with males exhibiting lower locomotion.
- tACS effects seem to be sex- and frequency-dependent, which is critical for clinical protocols.

## Background

Sex and gender differences are important issues in various neuropsychiatric disorders [1]. Significant differences in incidence, age of onset, and clinical symptoms have been observed between males and females. For example, around two-thirds of Alzheimer’s disease (AD) patients are women, a trend that cannot be solely attributed to their longer life expectancy [2]. These sex differences may be caused by various factors, including sex hormones, astrocytes, microglia, placental sex, and epigenetic modifications [1, 3]. However, the role of sex differences has long been overlooked in the development of drug and non-drug therapies [3].

Transcranial electrical stimulation (tES) is a promising, noninvasive neuromodulation strategy for enhancing cognitive function in both neuropsychiatric patients and healthy individuals [4–7]. It has two main forms: transcranial direct current stimulation (tDCS) and transcranial alternating current stimulation (tACS). tDCS modulates cortical excitability by regulating the resting membrane potential of neurons [8], while tACS influences neural activity by entraining endogenous rhythms at specific frequencies [9]. Notably, the effects of tDCS vary by sex [10, 11]. For example, studies have demonstrated stronger effects in females regarding decision-making, social cognition, and neuropsychiatric disorders [12–15], while more pronounced effects have been observed in males regarding attention and sleep [16, 17]. In addition, sex-specific patterns have emerged in hemisphere sensitivity and glutamate modulation [18, 19]. However, the sex-specific effects of tACS have not been widely explored, despite its unique oscillatory mechanisms.

The principle of tACS is to use exogenous rhythms to regulate endogenous brain rhythms, which can be measured by electroencephalogram (EEG) oscillations in different frequency bands [9]. These oscillations have been suggested to exhibit sex-related differences in human subjects. For example, females show higher beta activity than males of the same age during childhood and adolescence [20]. Among athletes, females exhibit greater beta activity in the prefrontal and temporal regions, as well as higher theta activity in the parietal and occipital regions, than males do [21]. In addition, robust sex differences in resting-state EEG oscillations are evident in older adults (ages 55–80). Compared to females, males exhibit increased low alpha activity in the temporal regions and decreased low beta activity in the parietal-occipital areas [22]. These sex-related variations in EEG oscillations across different frequency bands support the possibility that tACS may have frequency-specific effects on males and females.

This study investigated whether tACS has sex-dependent effects on the cognitive behavior of male and female mice. Frequency is the most important feature of tACS. The most commonly used frequencies in published tACS studies are gamma frequencies, such as 40 Hz, followed by alpha frequencies, such as 10 Hz [23]. These frequencies are also commonly used to improve cognitive function in healthy, aging, and psychiatric populations [7]. Another important feature of tACS is the target area. In human studies, the primary target is the dorsolateral prefrontal cortex (DLPFC) [7, 23]. When single-site tACS is used, the left region is often targeted [7]. Consistent with these findings, the present study examined sex-related differences in cognition induced by tACS at 10 and 40 Hz by targeting the left prefrontal cortex (PFC) of mice. This study may help optimize tACS parameters for translational research.

## Methods

### Animals

**Fig. 1:**
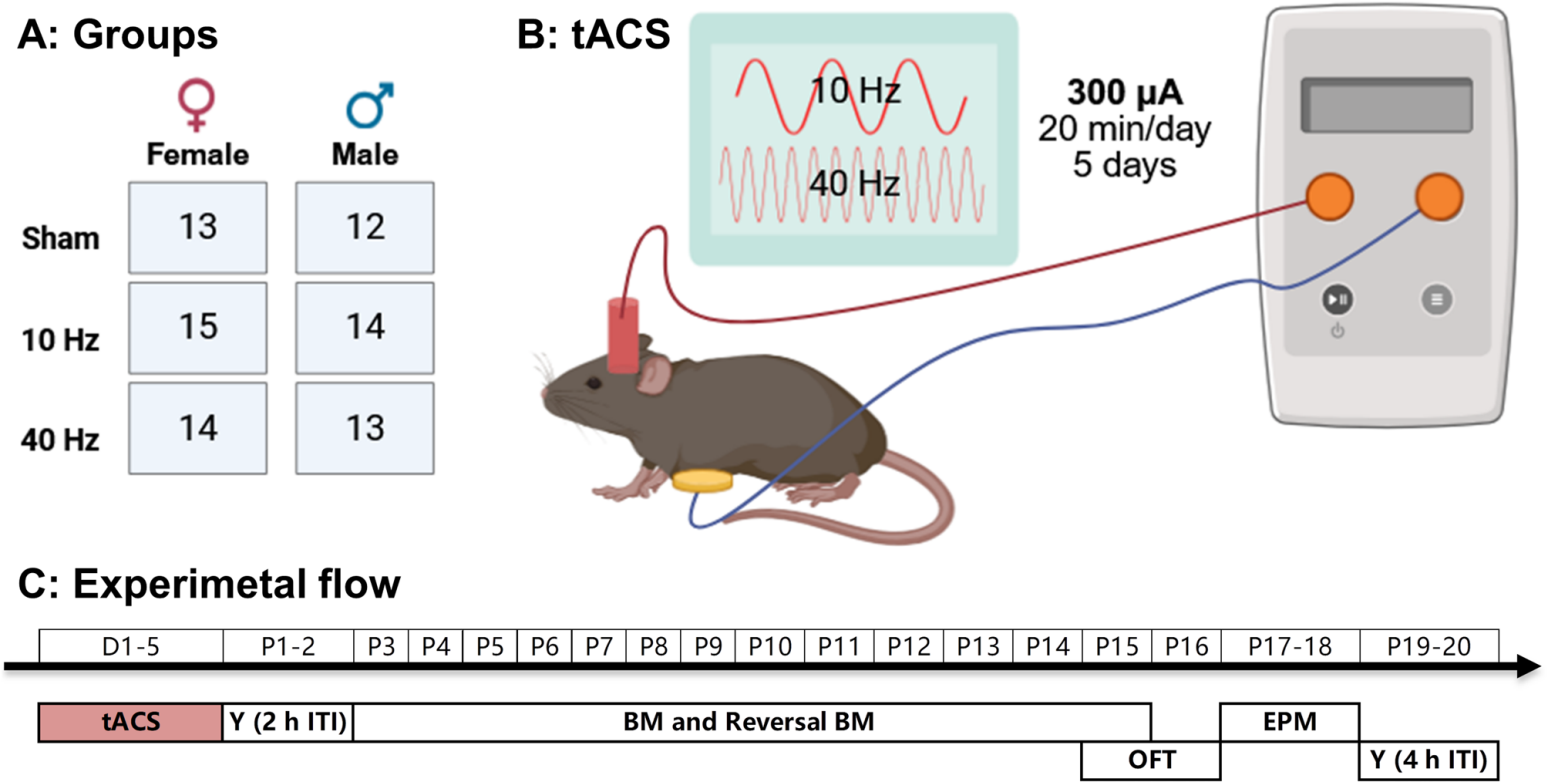
Experimental design. (**A**) Number of mice in each tACS group categorized by sex. (**B**) Each mouse was implanted with a plastic tube on the skull that was filled with saline and used as a tACS electrode. The other electrode was placed on the mouse’s abdomen. (**C**) Experiment flow: D1-D5 and P1-P20 represent the days of tACS and the days after tACS, respectively. Y represents the Y-maze task, BM represents the Barnes maze task, OFT represents the open-field task, and EPM represents the elevated plus maze task. Created in BioRender. Fu, Y. (2025) https://BioRender.com/1gvaicv

A total of 81 four-month-old C57BL/6 mice were used in this study. Of these, 42 were female and 39 were male. The animals were randomly divided into three tACS groups by sex. Please refer to Fig. 1A for the specific number of mice in each group. The mice were housed in a temperature- and humidity-controlled environment that followed the natural light-dark cycle. They had free access to food and water. All laboratory and animal welfare procedures were conducted in accordance with national guidelines and were approved by the National Animal Research Ethics Committee. In addition, the Animal Care and Use Committee of Kunming University of Science and Technology approved all experimental procedures.

## Experiment schedule

The animals underwent surgery to install an epicranial plastic tube for tACS (Fig. 1B). After the procedure, the animals were given at least one week to recover. Prior to tACS, the animals had 3 days to adapt to the stimulation conditions. Then, tACS was performed continuously for 5 days. The following paradigms were performed sequentially to test the effects of tACS on spatial cognition and general behaviors (Fig. 1C). (1) The Y-maze task, which included two trials (training and testing), was used to test spatial recognition memory. Previous studies have shown that this type of memory lasts no longer than a few hours [24]. Therefore, the intertrial interval (ITI) was set at 2 h and 4 h, representing moderate and high memory difficulty levels, respectively. (2) The Barnes maze (BM) task assesses spatial learning and memory through its training phase (acquisition phase) and probe trial, while the reversal BM task evaluates cognitive flexibility through reversal learning trials [25]. (3) The open-field test (OFT) assessed locomotion, exploration and general-anxiety behaviors. (4) The elevated plus maze (EPM) was used to test anxiety levels. The timing schedule for each paradigm relative to tACS is shown in the results of each figure.

## Transcranial alternating current stimulation

Before tACS, a plastic tube was installed on the skull of the left PFC of each animal. The animals were anesthetized with an intraperitoneal (i.p.) injection of 80 mg/kg of sodium pentobarbital (10 mg/mL; dissolved in saline; Merck). Local anesthesia was administered by subcutaneously injecting a lidocaine solution (0.2 mL; Shanghai Pujin Linzhou Pharmaceutical Co., Ltd.) into the scalp. Then, an incision was made in the scalp to expose and clean the skull. Using a stereotaxic device, the coordinates of the left PFC were marked: +2.95 mm for AP and − 1.5 mm for ML. Next, a round plastic tube with an inner diameter of 3.6 mm was placed at the center of the marked location on the skull and secured with AB glue. Three small screws (M1.0 × L2.0 mm; Shenzhen RWD Life Science Co., Ltd.) were installed on the skull for fixation. Finally, dental cement was poured to secure the tube and screws. The animals rested for at least 7 days.

The tACS procedure was consistent with our previous work [26, 27]. In brief, a 3-day adaptation period was required before tACS. On the first day, the animals were placed on the stimulation platform for 15 min to allow them to become familiar with it. On the second day, the animals were restrained on the platform for 5 min to acclimate them to restraint. On the third day, they were restrained for 10 min. After the adaptation period, the animals received 5 tACS treatments (Cerebooster, Droian, Hangzhou Zhuo’anzi Network Technology Co., Ltd.), once daily for 20 min each. A sponge soaked in saline solution was placed in the tACS plastic tube, and conductive gel was applied to their abdomens. The sponge and gel served as the active electrodes for tACS. The animals were kept awake and restrained during tACS and monitored in real time. tACS consisted of sinusoidal alternating current stimulation at 10–40 Hz with a density of 2.95 mA/cm^2^ (300 µA/0.1017 cm^2^). The sham group underwent the same procedure as the stimulation groups but without electrical stimulation.

## Behavioral paradigms

All behavioral tasks are monitored in real time by a software (KEmaze, KEW Basis, China), which can simultaneously capture and analyze the movement trajectories of one or more animals.

The Y-maze task used in this study is a two-trial task that does not involve rewards or punishments [24]. The maze consists of three arms: novel, starting and other, each measuring 8 × 15 × 30 cm (width × height × length). There were no visual cues inside the maze. The task had 2 trials: a training trial and a testing trial, with an ITI of 2–4 h between them. In the training trial, the novel arm is blocked, leaving the other two arms open. Each animal was placed in the starting arm and allowed to freely explore for 10 min. In the testing trial, the novel arm was open and the animal was allowed to explore for 5 min. The positions of the three arms were randomly assigned to each animal and balanced within each tACS group. To avoid stress caused by handling the mice, data from the starting arm were excluded from the statistical analysis. For each animal, the time spent in the novel and other arms, the number of visits, and walking distance (ambulation) were calculated as percentages. Higher percentages of these indices in the novel arm indicate better spatial recognition memory.

The BM task utilizes a circular platform (diameter: 90 cm) positioned 90 cm above the ground, with 20 holes (diameter: 5 cm) evenly distributed on the platform. The task comprises 3 phases: adaptation, training, and testing. During the adaptation phase (day 1), animals were habituated to escape rules. A random hole was designated as the escape chamber, and two bright lights were turned on as aversive stimuli. Animals placed at the platform’s center were guided to enter the escape chamber, after which the lights were turned off and the animals were housed in the chamber for 2 min. During the training phase (days 2–5), each animal was assigned a random hole as the target hole. The animal underwent 3 trials daily, with a 15-minute ITI. In each trial, the animal was placed at the platform’s center, the lights were turned on, and the animal was allowed to freely explore. If the animal found the target hole and fully entered the escape chamber within 3 min, it rested in the chamber for 30 s. If not, it was guided into the chamber and rested for 1 min. During the testing phase (day 6; probe trial), the escape chamber was removed. The mouse was placed at the platform’s center, the lights were turned on, and the animal was allowed to freely explore for 90 s. The testing phase was performed 24 h after the final trial of the training phase. Data collection included: the escape latency to find the target hole in each training trial, the escape latency to find the target hole for first time in the probe trial, and the number of nose-pokes to each hole in the probe trial (TG: the target hole; OH: the hole opposite the target; L1-9: the left holes of the target; and R1-9: the right holes of the target). In addition, the speed and total distance were recorded for the probe trial.

For each animal, the OH used in the above BM served as the new target hole for the reversal BM. In other words, the target hole was reversed by 180°. The reversal BM has two phases: training and testing. All procedures and data collection in both phases were identical to those in the BM.

The OFT was performed in a square box, measuring 50 × 50 × 30 cm (length × width × height). The floor of the OFT was divided into sixteen squares. The four central squares formed the central area and the remaining squares formed the peripheral area. During the test, each animal was placed in the box and allowed to explore freely for 10 min. The following were recorded: ambulation distance and time spent in the central and peripheral areas, rearing times, and fecal droppings. Higher percentages of ambulation distance and time spent in the central area indicate less anxiety.

The EPM consisted of two closed arms and two open arms. Each arm measures 6 × 15 × 30 cm (width × height × length). During the test, each animal was placed in one of the open arms and allowed to explore freely for 5 min. For each animal, the time spent in each arm, the number of visits, and the ambulation distance were calculated as percentages. Higher percentages of these indices in the open arms indicate less anxiety. In addition, dipping times and rearing times, as well as the total number of arm visits and total ambulation distance were recorded for each animal.

### Statistical analysis

SPSS was used for statistical analysis and GraphPad Prism for data visualization. Data normality was first assessed using the Shapiro-Wilk test. For normally distributed data, a two-way ANOVA (ANOVA-2) was used to assess the main effects of sex and frequency, as well as their interaction. In addition, a one-way ANOVA (ANOVA-1) was used to assess frequency differences within each sex, and an independent-samples t-test was used to assess sex differences within each frequency. A repeated-measures ANOVA (ANOVA-R) was used to assess indices that changed over time (e.g., latencies to find the target in the BM test). For non-normally distributed data, the Kruskal-Wallis test was used to assess frequency differences within each sex, and the Mann-Whitney U test was used to assess sex differences within each frequency. The Friedman test was used to assess indices that changed over time. Furthermore, to determine whether indices (e.g., novel arm percentages in the Y-maze test) were significantly higher than the chance level, a one-sample t-test was used for normally distributed data, and the Wilcoxon signed-rank test was used for non-normally distributed data. Unless stated otherwise, data are presented as the mean ± standard error of the mean (SEM). Statistical significance was set at *P* < 0.05, with specified statistical values were noted in the results sections.

## Results

### 40 Hz tACS improves spatial recognition memory in male mice

**Fig. 2:**
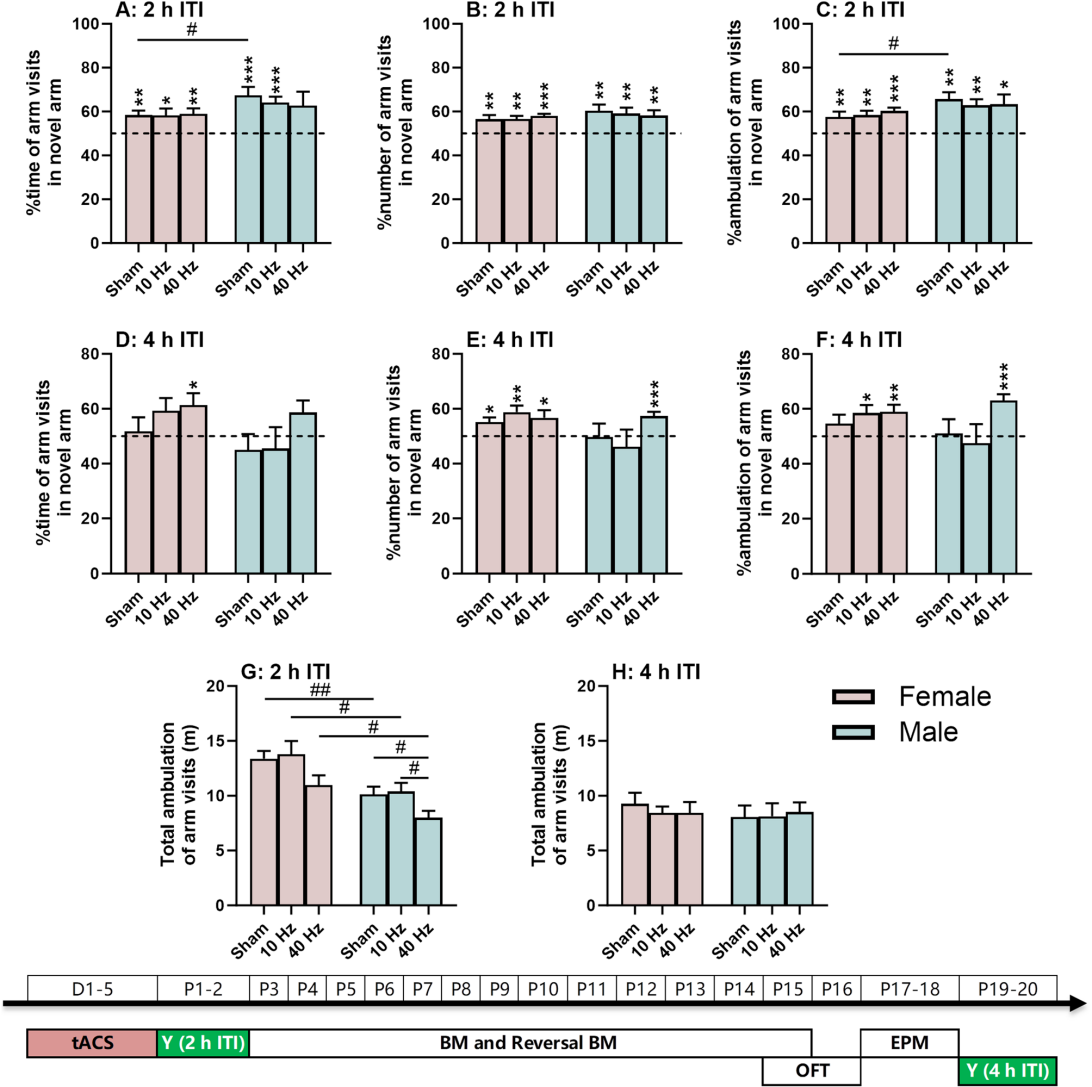
Spatial recognition memory based on the exploratory nature of the animal. (**A**-**C**) Y-maze task with a 2-h inter-trial interval (ITI). (**D**-**F**) Y-maze task with a 4-h ITI. (**G**-**H**) Total ambulation distance in the maze as a locomotor index. The experimental times are shown below the figures in white text on a green background. The (*) symbol indicates significant differences compared to the 50% chance level (dashed line). The (#) symbol indicates post-hoc significant differences between groups. One symbol denotes *P* < 0.05, two symbols denote *P* < 0.01, and three symbols denote *P* < 0.001

Spatial recognition memory was assessed with a two-trial Y-maze task. When the intertrial interval was short (2 h ITI; Fig. 2A-C), females and males in all three tACS frequency groups discriminated the novel from the other arms. The percentages of time, number of arm visits, and/or ambulation in the novel arm were significantly higher than the chance level of 50% (*P* < 0.05 for all, see figures for detailed significance; the same below). Interestingly, at this intertrial interval, females in the sham group had poorer memory than males, with significantly lower percentages of time (F(1, 75) = 4.200, *P* < 0.05 for the main effect of sex; t(23) = −2.089, *P* < 0.05 for females vs. males) and ambulation (U = 119.000, *P* < 0.05 for females vs. males) in the novel arm than males.

When the intertrial interval was long (4 h ITI; Fig. 2D-F), females in all three tACS frequency groups also showed good discrimination for the novel arm. At least one of the three indices was significantly higher than chance (*P* < 0.05 for all). In males, however, only the 40 Hz tACS group was able to discriminate the novel from the other arms, with significantly higher percentages of arm visits and ambulation in this arm than chance (t(23) = 4.723 and 5.692 for number and ambulation, respectively, *P* < 0.001 for both; Fig. 2E-F). In contrast, both the sham and 10 Hz tACS groups failed to detect the novel arm (*P* > 0.05 for all).

Locomotion for this task was assessed by total ambulation in the maze (Fig. 2G-H). This index showed significant main effects of sex (F(1, 75) = 20.268, *P* < 0.001) and frequency (F(2, 75) = 5.355, *P* < 0.01). In all three tACS groups, females had higher ambulation than males (sham: t(23) = 3.245, *P* < 0.01; 10 Hz: t(27) = 2.326, *P* < 0.05; 40 Hz: t(25) = 2.683, *P* < 0.05; Fig. 2G). In addition, in males, the 40 Hz tACS group had a lower ambulation than the other two groups (F(2, 36) = 3.381, *P* < 0.05 for the main effect of frequency; *P* < 0.05 for 40 Hz vs. the other two groups). These effects were observed only for the 2-h ITI and not for the 4-h ITI tasks (*P* > 0.05 for all; Fig. 2H).

### 10 Hz and 40 Hz tACS enhances spatial learning in female mice

**Fig. 3:**
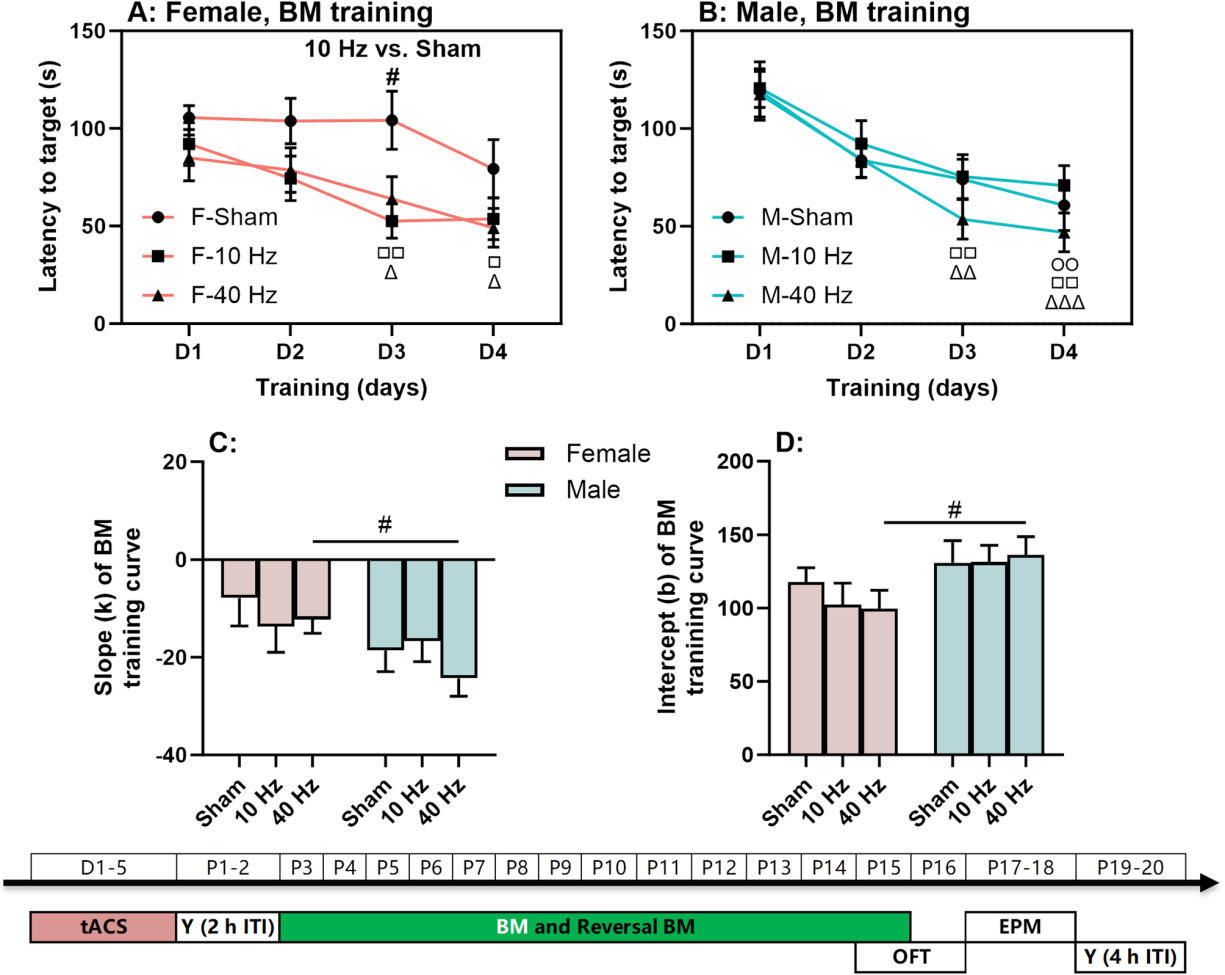
Spatial learning with aversive bright light as punishment. (**A**) Barnes maze (BM) training curves in females. (**B**) BM training curves in males. (**C**-**D**) Slopes and intercepts of training curves in females and males. The experimental times are shown below the figures in white text on a green background. The symbols(○, □, and △) indicate significant differences compared to Day 1 (D1) for the Sham, 10 Hz tACS, and 40 Hz tACS groups, respectively. The (#) symbol indicates significant differences between groups. Post-hoc symbols are shown. One symbol denotes *P* < 0.05, two symbols denote *P* < 0.01, and three symbols denote *P* < 0.001

Spatial learning was assessed using the BM training task (Fig. 3). In females (Fig. 3A), both 10 Hz and 40 Hz tACS groups showed significantly decreased latencies to find the target hole across training days (10 Hz: χ^2^ = 14.120, *P* < 0.01; 40 Hz: χ^2^ = 15.086, *P* < 0.01). In both groups, latencies on days 3–4 were significantly lower than those on day 1 (*P* < 0.05 for all from pairwise comparisons, see figures for detailed significance; the same below). In contrast, the sham group showed no significant decrease in latencies over time (F(3, 36) = 1.569, *P* = 0.214). In addition, on day 3, the 10 Hz tACS group showed significantly more decreased latencies than the sham group (H = 7.616, *P* < 0.05 for the main effect of frequency; *P* < 0.05 for 10 Hz vs. sham). In males (Fig. 3B), all three tACS groups showed significantly decreased latencies to find the target hole over time (sham: χ^2^ = 13.900, *P* < 0.01; 10 Hz: F(3, 39) = 5.515, *P* < 0.01; 40 Hz: χ^2^ = 26.814, *P* < 0.001). In all groups, latencies on day 3 and/or 4 were significantly lower than those on day 1 (*P* < 0.05 for all from pairwise comparisons).

For all training curves, slopes and intercepts were calculated for comparison between sexes and between tACS frequencies (Fig. 3C-D). A sex difference was observed in the 40 Hz tACS group. Males had a significantly lower slope than females (U = 42.000, *P* < 0.05), indicating faster learning speeds. In addition, females had significantly lower intercept than males (U = 135.000, *P* < 0.05), suggesting better initial training performance.

## tACS has little effect on spatial reversal learning (cognitive flexibility) in either sex

**Fig. 4:**
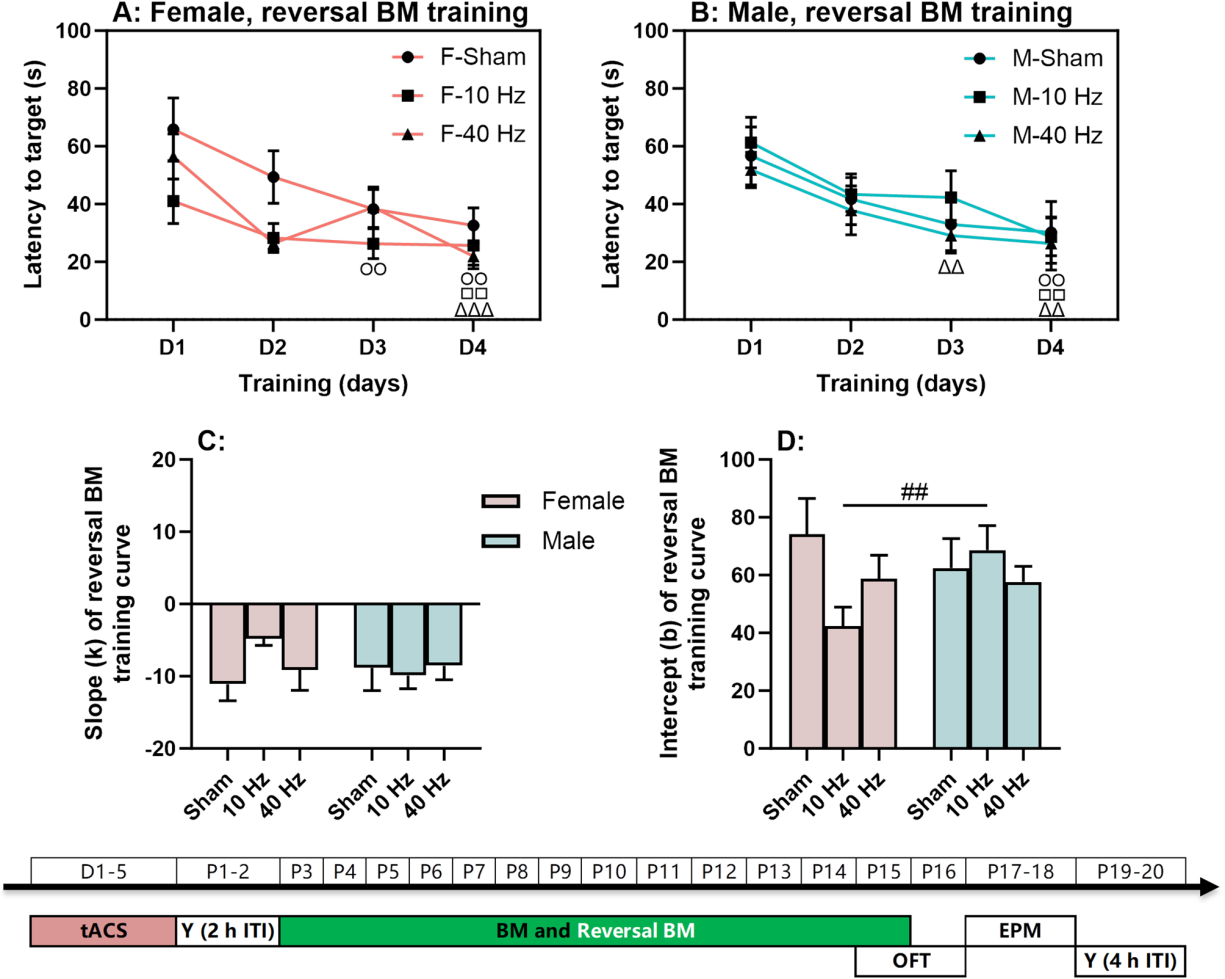
Spatial reversal learning with aversive bright light as punishment. (**A**) Reversal Barnes maze (BM) training curves in females. (**B**) Reversal BM training curves in males. (**C**-**D**) Slopes and intercepts of the reversal training curves in females and males. The experimental times are shown below the figures in white text on a green background. The symbols(○, □, and △) indicate significant differences compared to Day 1 (D1) for the Sham, 10 Hz tACS, and 40 Hz tACS groups, respectively. The (#) symbol indicates significant differences between groups. Post-hoc symbols are shown. One symbol denotes *P* < 0.05, two symbols denote *P* < 0.01, and three symbols denote *P* < 0.001

Spatial reversal learning was assessed using the reversal BM training task (Fig. 4). All three tACS groups showed significantly reduced latencies to find the target hole in both females (sham: χ^2^ = 17.031, *P* < 0.01; 10 Hz: χ^2^ = 13.640, *P* < 0.01; 40 Hz: χ^2^ = 18.943, *P* < 0.001; Fig. 4A) and males (sham: χ^2^ = 10.400, *P* < 0.05; 10 Hz: χ^2^ = 20.180, *P* < 0.001; 40 Hz: χ^2^ = 18.256, *P* < 0.001; Fig. 4B) over the reversal training days. In all groups, latencies on day 3 and/or day 4 were significantly lower than those on day 1 (*P* < 0.05 for all from pairwise comparisons).

Slopes and intercepts of the training curves were also calculated for females and males (Fig. 4C-D). The slopes showed no significant difference between sexes and tACS frequencies (*P* > 0.05 for all). A sex difference was observed in the 10 Hz tACS group, females had a significantly lower intercept than males (U = 169.500, *P* < 0.01), suggesting better initial training performance.

## tACS has little effect on spatial memory in either sex

**Fig. 5:**
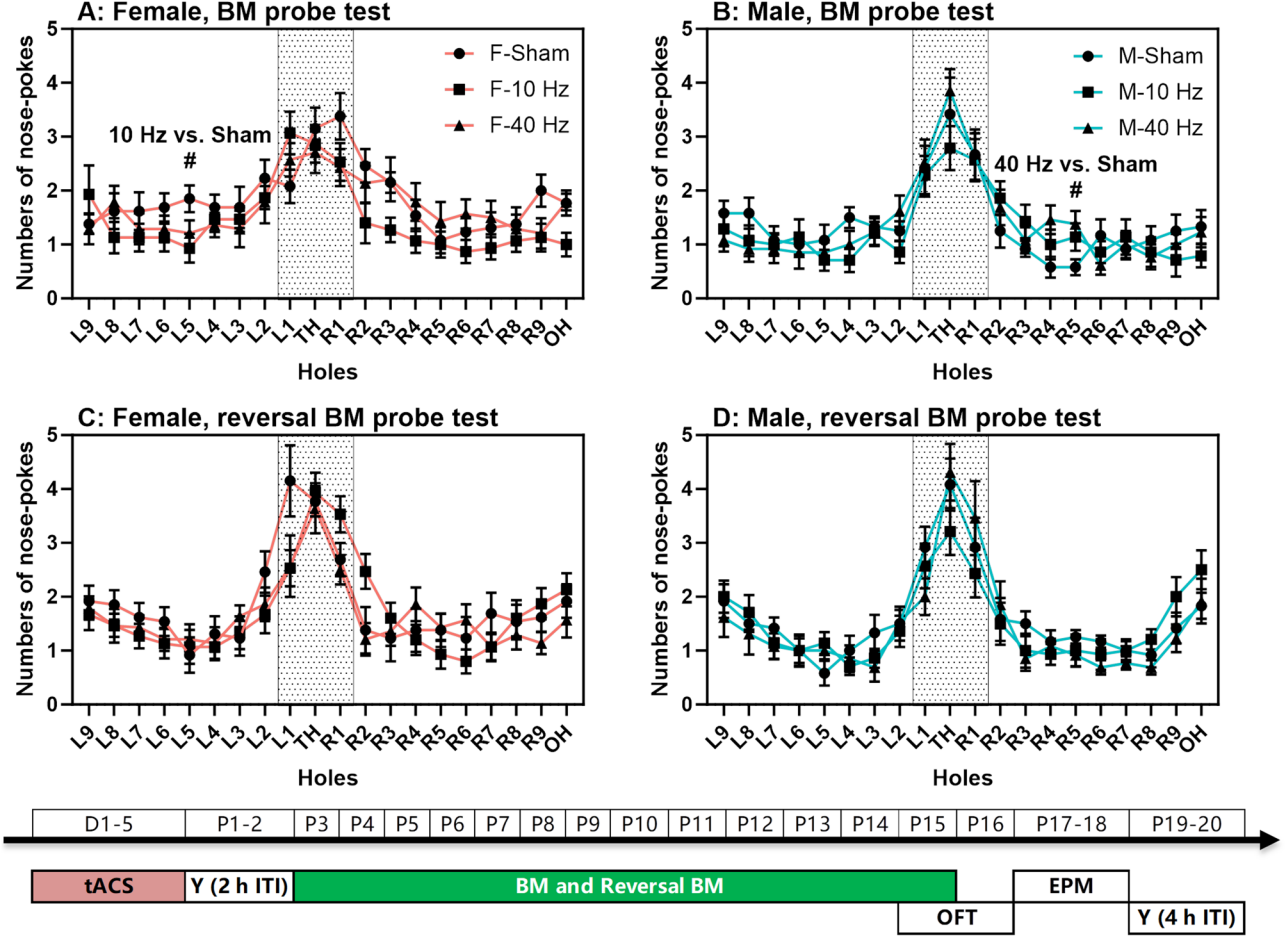
Spatial memory with aversive bright light as punishment. (**A**-**B**) Numbers of nose-pokes to all holes during the Barnes maze (BM) probe test are shown for females and males, respectively. (**C**-**D**) Numbers of nose-pokes to all holes during the reversal BM probe test are shown for females and males, respectively. The experimental times are shown below the figures in white text on a green background. The (#) symbol indicates post-hoc significant differences at *P* < 0.05 between groups

Spatial memory was assessed using probe tests after BM and reversal BM training. Females and males in all three groups showed more nose-pokes in the target areas (the target hole and the two holes to its left and right; Fig. 5). There were no significant differences in this index or latencies to find the target hole between sexes or tACS frequencies (*P* > 0.05 for all; Table 1).

**Table 1:** Effects of tACS on the indices of the Barnes maze and reversal Barnes maze tests in females and males

Tasks (indices)		Female			Male			Sex difference
		Mean	SD	*N*	Mean	SD	*N*	
***Barnes maze (BM)***								
**Number of nose-pokes in target area**	Sham	8.615	3.453	13	8.50	3.90	12	
	10 Hz	8.467	2.997	15	7.64	2.79	14	
	40 Hz	7.714	3.197	14	9.00	3.27	13	
**Test latency (s)**	Sham	15.923	18.495	13	15.92	9.32	12	
	10 Hz	22.467	27.089	15	13.21	11.03	14	
	40 Hz	29.714	28.886	14	18.46	20.65	13	
**Total number of nose-pokes**	Sham	37.308	14.744	13	28.00	6.47	12	
	10 Hz	29.267	6.352	15	25.43	6.60	14	
	40 Hz	33.929	10.731	14	27.69	6.45	13	
**Speed (mm/s)**	Sham	91.068	23.394	13	85.73	14.17	12	
	10 Hz	**90.781**	**13.159**	**15**	**74.35**	**17.90**	**14**	***P = 0.009*** ^**##**^
	40 Hz	**100.552**	**35.002**	**14**	**78.68**	**13.27**	**13**	***P= 0.022*** ^**#**^
***Reversal Barnes maze (reversal BM)***								
**Number of nose-pokes in target area**	Sham	10.615	2.873	13	9.917	3.801	12	
	10 Hz	10.000	3.229	15	8.214	3.867	14	
	40 Hz	8.714	3.474	14	9.769	4.246	13	
**Test latency (s)**	Sham	14.846	13.643	13	15.250	14.162	12	
	10 Hz	16.533	13.658	15	13.214	8.469	14	
	40 Hz	15.357	11.257	14	12.154	8.934	13	
**Total number of nose-pokes**	Sham	36.846	15.614	13	32.000	7.508	12	
	10 Hz	34.333	14.075	15	30.214	9.316	14	
	40 Hz	32.857	10.235	14	28.846	9.651	13	
**Speed (mm/s)**	Sham	101.549	28.897	13	90.217	12.123	12	
	10 Hz	**96.939**	**25.665**	**15**	**82.935**	**10.383**	**14**	***P = 0.041*** ^**#**^
	40 Hz	100.949	25.318	14	86.766	12.031	13	

^#^*P* < 0.05 and ^##^*P* < 0.01 for significant and highly significant differences between females and males, respectively. Significant indices are highlighted in bold.

All indices: No significance was found between the three tACS frequency groups

In the BM probe test, females had significantly higher speeds than males in the 10 Hz and 40 Hz tACS groups (10 Hz: t(27) = 2.830, *P* < 0.01; 40 Hz: U = 44.000, *P* < 0.05; Table 1). In the reversal BM probe test, females had significantly higher speeds than males in the 10 Hz tACS group (U = 58.000, *P* < 0.05; Table 1). Additionally, there was no significant difference in the total number of nose-pokes between sexes or tACS frequencies in either test (*P* > 0.05 for all).

### tACS has little effect on anxiety levels in either sex

Locomotor activity and general anxiety were measured using the OFT test (Table 2). Total ambulation, percentages of time and ambulation in the central area, number of rearing times, and number of fecal droppings showed no significant difference between sexes and between tACS frequencies (*P* > 0.05 for all). Anxiety behavior was further assessed using the EPM test (Table 2). The percentages of time, ambulation, and number of arm visits in open arms also showed no significant difference between sexes and between tACS frequencies (*P* > 0.05 for all), as did the number of dipping times and rearing times (*P* > 0.05 for all).

**Table 2:** Effects of tACS on open-field and elevated plus maze indices in females and males

Tasks (indices)		Female			Male			Sex difference
		Mean	SD	*N*	Mean	SD	*N*	
***Open-field test (OFT)***								
**Ambulation (m)**	Sham	54.030	16.049	13	45.530	9.288	12	
	10 Hz	47.650	13.151	15	48.624	12.369	14	
	40 Hz	49.605	6.463	14	48.685	8.810	13	
**% time in center**	Sham	4.816	2.124	13	5.661	2.406	12	
	10 Hz	5.895	3.428	15	5.455	2.925	14	
	40 Hz	5.474	4.302	14	5.471	2.671	13	
**% ambulation in center**	Sham	10.549	3.686	13	11.161	2.929	12	
	10 Hz	10.932	4.824	15	10.731	3.365	14	
	40 Hz	11.028	5.220	14	11.596	5.064	13	
**Rearing times**	Sham	91.769	20.588	13	81.333	18.884	12	
	10 Hz	79.400	29.154	15	83.571	22.991	14	
	40 Hz	93.071	19.121	14	91.154	17.272	13	
**Fecal counts**	Sham	0.539	0.877	13	0.167	0.389	12	
	10 Hz	1.333	1.447	15	0.571	1.016	14	
	40 Hz	1.214	1.762	14	0.462	0.776	13	
***Elevated plus maze (EPM)***								
**% time in open arms**	Sham	43.854	7.548	13	45.315	13.678	12	
	10 Hz	41.283	10.367	15	46.741	11.018	14	
	40 Hz	47.200	12.320	14	44.783	14.851	13	
**% ambulation in open arms**	Sham	39.213	8.375	13	40.471	10.837	12	
	10 Hz	36.260	10.748	15	39.992	11.565	14	
	40 Hz	41.355	9.455	14	36.943	9.934	13	
**% arm visits in open arms**	Sham	42.735	8.755	13	43.870	9.392	12	
	10 Hz	43.403	8.938	15	43.914	8.969	14	
	40 Hz	44.219	9.210	14	41.995	9.519	13	
**Dipping times**	Sham	37.923	8.883	13	38.167	6.820	12	
	10 Hz	33.533	7.689	15	37.929	7.711	14	
	40 Hz	35.857	7.912	14	32.000	7.757	13	
**Rearing times**	Sham	21.308	8.107	13	15.667	6.050	12	
	10 Hz	19.000	5.593	15	18.214	12.217	14	
	40 Hz	17.857	6.062	14	14.154	6.890	13	
**Total number of arm visits**	Sham	**55.385**	**8.809**	**13**	**46.583**	**9.774**	**12**	***P = 0.027*** ^**#**^
	10 Hz	52.067	11.183	15	45.214	8.772	14	
	40 Hz	50.571	9.196	14	44.231	10.224	13	
**Ambulation (m)**	Sham	**15.551**	**1.681**	**13**	**12.407**	**1.817**	**12**	***P*** ***< 0.001***^**###**^
	10 Hz	14.097	2.778	15	12.400	2.200	14	
	40 Hz	**14.030**	**2.635**	**14**	**11.701**	**2.271**	**13**	***P = 0.021*** ^**#**^

^#^*P* < 0.05 and ^###^*P* < 0.01 for significant and very highly significant differences between females and males, respectively. Significant indices are highlighted in bold.

All indices: No significance was found between the three tACS frequency groups

For locomotion, females in the sham group had a significantly higher total number of arm visits than males in the EPM test (F(1, 75) = 11.460, *P* < 0.01 for the main effect of sex; t(23) = 2.368, *P* < 0.001 for females vs. males; Table 2). In addition, females in the sham and 40 Hz tACS groups had a higher ambulation than males (F(1, 75) = 21.950, *P* < 0.001 for the main effect of sex; females vs. males: t(23) = 4.495, *P* < 0.001 for sham, and t(25) = 2.451, *P* < 0.05 for 40 Hz).

### Summary of the effects of tACS on locomotion in female and male mice

The above tests revealed significant sex differences in locomotion in both the tACS and sham groups. To determine whether these differences were due to sex or tACS, we compared the normalized values in Table 3. For both females and males, locomotion was lower in the 10-Hz and 40-Hz tACS groups than in the sham group. In addition, males exhibited lower locomotion than females (see the table for details). Therefore, both sex and tACS are responsible for the observed differences in locomotion, with tACS potentially amplifying inherent sex-related disparities.

**Table 3:** Summary of the effect of tACS on locomotion in females and males

Tasks	Locomotor indices	Sham *n*_f_=13 *n*_m_=12	10 Hz *n*_f_=15 *n*_m_=14	40 Hz *n*_f_=14 *n*_m_=13	(10 Hz-Sham) /Sham*100	(40 Hz-Sham) /Sham*100
**Females**					***−4.589*** ^a^	***−5.226*** ^a^
Y: 2 h ITI	Ambulation (m)	13.375	13.786	10.970	3.070	*−17.984* ^a^
OFT	Ambulation (m)	54.030	47.650	49.605	*−11.808* ^a^	*−8.190* ^a^
EPM	Ambulation (m)	15.551	14.097	14.030	*−9.350* ^a^	*−9.781* ^a^
BM	Speed (mm/s)	91.068	90.781	100.552	*−0.315* ^a^	10.414
Reversal BM	Speed (mm/s)	101.549	96.939	100.949	*−4.540* ^a^	*−0.591* ^a^
**Males**					**−2.370** ^**a**^	**−6.374 ** ^**a**^
Y: 2 h ITI	Ambulation (m)	10.117	10.396	7.986	2.758	*−21.059* ^a^
OFT	Ambulation (m)	45.530	48.624	48.685	6.796	6.929
EPM	Ambulation (m)	12.407	12.400	11.701	*−0.056* ^a^	*−5.690* ^a^
BM	Speed (mm/s)	85.730	74.350	78.680	*−13.274* ^a^	*−8.223* ^a^
Reversal BM	Speed (mm/s)	90.217	82.935	86.766	*−8.072*	*−3.825*
(Males-Females)/Females*100					**85.731** ^**c**^	**202.537** ^**c**^
Y: 2 h ITI	Ambulation (m)	*−24.363* ^b^	*−24.592* ^b^	*−27.200* ^b^	*0.940* ^c^	*11.641* ^c^
OFT	Ambulation (m)	*−15.732* ^b^	2.044	*−1.855* ^b^	−112.993	−88.211
EPM	Ambulation (m)	*−20.217* ^b^	*−12.038* ^b^	*−16.600* ^b^	−40.457	−17.892
BM	Speed (mm/s)	*−5.862* ^b^	*−18.100* ^b^	*−21.752* ^b^	*208.785* ^c^	*271.095* ^c^
Reversal BM	Speed (mm/s)	*−11.159* ^b^	*−14.446* ^b^	*−14.050* ^b^	*29.456* ^c^	*25.903* ^c^

n_f_ and n_m_: Number of females and males in each tACS group, respectively

Bold values: The averaged changes calculated from the 5 normalized values below the value

^a^**:**Underlined negative values indicate decreased locomotion in the tACS frequency group relative to the sham group

^b^**: **Underlined negative values indicate decreased locomotion in the males relative to females

^c^**: **Underlined positive values indicate decreased locomotion in the tACS frequency group relative to the sham group

## Discussion

This study found that tACS improved spatial recognition memory in male mice at 40 Hz, and enhanced spatial learning in female mice at 10 and 40 Hz. In addition, male mice exhibited faster learning speeds, while female mice demonstrated better initial learning performance. However, tACS had little effect on spatial reversal learning, spatial memory (punishment-related) and anxiety in either sex. Notably, male mice exhibited lower locomotion than female mice in most tests, and tACS may have exacerbated this trend, particularly in male mice. These results suggest that 10 Hz and 40 Hz tACS have sex-specific effects on the cognitive behavior of mice.

To date, very few studies have explored sex differences in tACS. However, preliminary results suggest that such differences may exist. In human subjects, alpha-tACS targeting the left or right DLPFC increased alpha brain frequency, which disrupted performance in males and slightly improved performance in females [28]. In addition, after receiving alpha-tACS of the primary somatosensory cortex, males exhibited a positive correlation between pain empathy performance and empathic concern, though no such correlation was noted in females [29]. In contrast, substantial evidence has accumulated for another tES technique, tDCS. For example, in a cognitive training task, females showed greater improvement than males, and females responded differently to various types of tDCS over the left or right DLPFC, a phenomenon that was not observed in males [30]. Moreover, some studies have reinforced the notion that biological sex is a critical factor in studying tDCS effects [30–32]. Further, tDCS effects can be influenced by sex hormones. Its neuroregulatory effects vary across the menstrual cycle due to fluctuations in estrogen and progesterone levels, particularly in relation to cognitive control, neuroplasticity, and clinical intervention [11]. Notably, non-invasive brain stimulation methods, including tDCS, tACS, and TMS, may interact with sex/polypeptide hormones [11]. Thus, our findings on tACS align with existing research on tDCS in highlighting sex differences.

This study found that 40 Hz tACS improved spatial recognition memory in male mice and that 10 and 40 Hz tACS enhanced spatial learning in female mice. Neither frequency had an effect on spatial reversal learning or punishment-related spatial memory. In addition to the sex differences discussed above, these results align with the frequency- and task-specificity of tACS in cognitive functions [33, 34]. For example, theta-tACS (4–7 Hz) improves working memory, executive functions, and declarative memory; gamma-tACS (30–80 Hz) improves auditory and visual perception; and alpha- (8–13 Hz) or gamma-tACS may enhance attention [33]. Mechanistically, tACS modulates memory via oscillatory entrainment. Theta and gamma frequencies selectively engage networks linked to working and long-term memory [33–35]. This is consistent with the observed improvements in spatial recognition memory and learning. The lack of effects on reversal learning and punishment-related spatial memory further reflects this task specificity. Together, these findings underscore the importance of considering tACS in the context of frequency, network engagement, and task demands, reinforcing the necessity of standardized reporting of tACS methods [36–38].

Our findings showed sex-related differences in spatial cognition after tACS. Male mice learned the BM task faster, while female mice performed better initiallly. Interestingly, even with sham stimulation, male and female mice showed tendencies toward differences in these indices, though significant disparities only emerged after tACS. This aligns with a previous study [29], which found no difference in pain empathy performance between alpha tACS and sham tACS targeting the primary somatosensory cortex. However, in the alpha tACS group, male subjects’ pain empathy performance was positively correlated with their empathic concern. In addition, sex differences have been observed in spatial tasks related to visuospatial working memory, mental rotation abilities, and navigational strategies [39]. Among young adults, males performed better than females, with the latter relying more on egocentric strategies and the former on allocentric strategies. Sex differences also appear in specific factors and tasks [40]. Males performed better than females on some, but not all, spatial tasks. However, females outperformed males on tasks requiring inhibition and attention control. Together, our data are consistent with previous reports on this point.

In the present study, male mice showed less locomotion than female mice in most behavioral tasks, and tACS appeared to amplify the difference between sexes. However, this observation is not supported by existing studies. The main difference is that we targeted the PFC, whereas these studies targeted the motor cortex or cerebellum. It has been shown that tACS may be a potential therapeutic tool to improve motor function or behavioral outcomes [41, 42]. Most tACS research focuses on its effects on motor control, cognitive function, and neuroplasticity, rather than examining sex-specific differences in locomotion. For example, cerebellar theta tACS reduces motor regularity [43], and beta tACS changes motor speed in Parkinson’s patients [44]. However, neither study examined sex differences. Similarly, studies on the time-dependent effects of cerebellar tACS on motor learning do not compare males and females [45]. Small sample sizes may be an important reason for this, or the study designs may not have considered sex as a factor. Further research is required to confirm these sex-dependent effects on locomotion, regardless of whether the stimulation targets the motor-related cortex or not.

Another finding of this study is that tACS had little effect on anxiety levels. This contrasts with previous research indicating that tACS can alleviate anxiety in neuropsychiatric disorders. For example, when the left DLPFC was targeted, tACS has been shown to be a more effective treatment for anxiety than tDCS in individuals with internalizing disorders [46]. In patients with anxiety disorders, tACS has produced notable average reductions in Beck Anxiety Inventory scores, as well as substantial symptom improvement in a large proportion of patients [47]. This study employed a novel tripod tACS protocol involving the placement of electrodes on the right frontal, parietal, and temporal cortices. Three factors may explain the discrepancy between these results and ours. First, the frequencies used differ: most literature reporting the anxiety-reducing effects of tACS has focused on theta-band stimulation (e.g., 5 Hz), whereas our study primarily used other frequencies (10 and 40 Hz). Second, tACS is thought to alleviate anxiety by modulating pathological neural oscillations and enhancing synchrony in emotion-regulation circuits [48, 49]. However, in healthy subjects or animals with intact emotion regulation circuits, there are no aberrant rhythms to correct, so tACS has no impact. This aligns with the state-dependent mechanisms of tACS [50]. Third, all of the mice in our study underwent restraint stress, which induces a transient but robust state of stress (e.g., elevated corticosterone). This differs from previous studies’ pathological anxiety states. This stress-mediated shift in baseline may have obscured the effects of tACS on anxiety, and alterations in brain oscillatory baselines could have reduced the effectiveness of our tested tACS frequencies in targeting anxiety-related circuits. Together, these findings highlight the importance of distinguishing the effects of tACS in healthy and pathological states, and accounting for stress-induced baseline shifts. Further research is needed to clarify anxiety modulation and how stress states interact with tACS parameters.

This study has several limitations. First, it lacks data on neural molecular mechanism, such as region-specific brain oscillation activity and molecular markers. This data is crucial for explaining how tACS drives sex-dependent cognitive effects. For example, microglia, which are influenced by estrogens in regulating inflammation, are a key target for studying sex differences in neurological disorders [51, 52]. In addition, tACS has been shown to decrease the diameter of microglia cell bodies in mice with AD [53]. Thus, future research should explore whether tACS interacts with microglia to mediate sex-dependent cognitive effects. Second, the range of tested tACS frequencies is limited. Since tACS effects are frequency-specific, a broader range of frequencies must be tested to understand potential sex-specific responses, such as theta and high-gamma frequency bands. Specifically, we did not include theta-band tACS (e.g., at a frequency of 5 Hz), a frequency commonly used in tACS studies [7]. We initially excluded theta tACS based on the pathophysiology of cognitive disorders, such as AD and mild cognitive impairment (MCI). These conditions exhibit pronounced sex differences and are characterized by EEG slowing including increases in delta and theta slow-wave activity [54, 55]. We hypothesized that entraining theta rhythms might exacerbate EEG slowing and worsen cognitive function. However, our recent findings in MCI model mice reveal significant off-frequency effects of tACS (unpublished data). It is possible that theta tACS enhances high-frequency activity (e.g., gamma) via mechanisms like cross-frequency coupling [38]. Since the animals in the present study were healthy, theta tACS could have provided valuable insights into sex-specific cognitive modulation. Third, tACS has only been tested on the left PFC. Although most tES in humans and animals targets the PFC [7, 23], there is still a lack of exploration into whether tACS-induced sex differences are specific to the PFC or exist across multiple brain regions. Future studies could expand the scope to other brain areas. Fourth, we did not measure or report corticosterone levels, even though restraint stress was used in the experimental protocol. This is a notable limitation because corticosterone assessments would have validated the stress state induced by restraint.

## Conclusions

In conclusion, this study reveals that tACS has sex- and frequency-dependent effects on spatial cognition in mice. These results highlight the importance of considering sex and stimulation frequency when optimizing tACS parameters for cognitive neurorehabilitation.

## Data Availability

The data are provided in the manuscript, and the corresponding author will make them available upon reasonable request.

## References

[1] Thibaut F. The role of sex and gender in neuropsychiatric disorders. Dialogues Clin Neurosci. 2016;18(4):351–2. 28179806 10.31887/DCNS.2016.18.4/fthibaut PMC5286720

[2] Association As. 2024 alzheimer’s disease facts and figures. Alzheimers Dement. 2024;20(5):3708–821. 38689398 10.1002/alz.13809 PMC11095490

[3] Lopez-Lee C, et al. Mechanisms of sex differences in Alzheimer’s disease. Neuron. 2024;112(8):1208–21. 38402606 10.1016/j.neuron.2024.01.024 PMC11076015

[4] Li Q, et al. Transcranial direct current stimulation of the dorsolateral prefrontal cortex for treatment of neuropsychiatric disorders. Front Behav Neurosci. 2022;16:893955. 35711693 10.3389/fnbeh.2022.893955 PMC9195619

[5] Liu A, et al. Immediate neurophysiological effects of transcranial electrical stimulation. Nat Commun. 2018;9(1):5092. 30504921 10.1038/s41467-018-07233-7 PMC6269428

[6] Yang C, Jung B, Lee SH. Transcranial electrical stimulation: clinical implication and practice for treatment of psychiatric illness. Clin Psychopharmacol Neurosci. 2024;22(3):391–404. 39069679 10.9758/cpn.23.1118 PMC11289600

[7] Grover S, et al. A meta-analysis suggests that tACS improves cognition in healthy, aging, and psychiatric populations. Sci Transl Med. 2023;15(697):eabo2044. 37224229 10.1126/scitranslmed.abo2044 PMC10860714

[8] Stagg CJ, Antal A, Nitsche MA. Physiology Transcranial Direct Curr Stimulation J Ect. 2018;34(3):144–52. 10.1097/YCT.0000000000000510 29877965

[9] Agnihotri SK, Cai J. Investigating the effects of transcranial alternating current stimulation on cortical oscillations and network dynamics. Brain Sci. 2024;14(8):767. 10.3390/brainsci14080767. 10.3390/brainsci14080767 PMC11353238 39199461

[10] Camchong J, et al. Frontal tDCS reduces alcohol relapse rates by increasing connections from left dorsolateral prefrontal cortex to addiction networks. Brain Stimul. 2023;16(4):1032–40. 37348702 10.1016/j.brs.2023.06.011 PMC10530485

[11] Veldema J. Non-invasive brain stimulation and sex/polypeptide hormones in reciprocal interactions: a systematic review. Biomedicines. 2023;11(7):1981. 10.3390/biomedicines11071981 37509620 10.3390/biomedicines11071981 PMC10377221

[12] Adenzato M, et al. Gender differences in cognitive theory of mind revealed by transcranial direct current stimulation on medial prefrontal cortex. Sci Rep. 2017;7:41219. 28117378 10.1038/srep41219 PMC5259730

[13] Balikshahi N, et al. Bihemispheric transcranial direct current stimulation over primary motor cortex potentiates improvement of neurological but not upper limb motor functions in ischemic stroke patients treated with routine physical therapy: a randomized, double-blind, sham-controlled trial. Am J Phys Med Rehabil. 2025;104(6):551–557. 10.1097/PHM.0000000000002669 39642344 10.1097/PHM.0000000000002669

[14] Fumagalli M, et al. Brain switches utilitarian behavior: does gender make the difference? PLoS ONE. 2010;5(1):e8865. 20111608 10.1371/journal.pone.0008865 PMC2810338

[15] He X, et al. Sex modulates the effect of HD-tDCS over the prefrontal cortex on the Iowa gambling task. Brain Stimul. 2023;16(2):415–7. 36731772 10.1016/j.brs.2023.01.1675

[16] Bakhshayesh Eghbali B, et al. Transcranial direct current stimulation improves sleep quality in patients with insomnia after traumatic brain injury. Brain Inj. 2023;37(1):63–73. 36408966 10.1080/02699052.2022.2145363

[17] de Tommaso M, et al. Effects of anodal TDCS stimulation of left parietal cortex on visual spatial attention tasks in men and women across menstrual cycle. Neurosci Lett. 2014;574:21–5. 24846414 10.1016/j.neulet.2014.05.014

[18] Meiron O, Lavidor M. Unilateral prefrontal direct current stimulation effects are modulated by working memory load and gender. Brain Stimul. 2013;6(3):440–7. 22743075 10.1016/j.brs.2012.05.014

[19] Mezger E, et al. Effects of bifrontal transcranial direct current stimulation on brain glutamate levels and resting state connectivity: multimodal MRI data for the cathodal stimulation site. Eur Arch Psychiatry Clin Neurosci. 2021;271(1):111–22. 32743758 10.1007/s00406-020-01177-0 PMC7867555

[20] Kavcic A, et al. Age related changes and sex related differences of functional brain networks in childhood: A high-density EEG study. Clin Neurophysiol. 2023;150:216–26. 37104911 10.1016/j.clinph.2023.03.357

[21] Tharawadeepimuk K, Nanbancha A, Onnom E. Characterizing psychological states in professional athletes through EEG: sex-based differences. EXCLI J. 2025;24:1–14. 39996238 10.17179/excli2024-7980 PMC11847956

[22] Han C, Cheung VCK, Chan RHM. Aging amplifies sex differences in low alpha and low beta EEG oscillations. Neuroimage. 2025;312:121231. 40252876 10.1016/j.neuroimage.2025.121231

[23] Gholamali Nezhad F, et al. Transcranial alternating current stimulation for neuropsychiatric disorders: a systematic review of treatment parameters and outcomes. Front Psychiatry. 2024;15:1419243. 39211537 10.3389/fpsyt.2024.1419243 PMC11360874

[24] Dellu F, et al. Genetic differences in response to novelty and spatial memory using a two-trial recognition task in mice. Neurobiol Learn Mem. 2000;73(1):31–48. 10686122 10.1006/nlme.1999.3919

[25] Gawel K, et al. Assessment of spatial learning and memory in the Barnes maze task in rodents-methodological consideration. Naunyn Schmiedebergs Arch Pharmacol. 2019;392(1):1–18. 30470917 10.1007/s00210-018-1589-y PMC6311199

[26] Duan M, et al. Anodal and cathodal transcranial direct current stimulations of prefrontal cortex in a rodent model of Alzheimer’s disease. Front Aging Neurosci. 2022;14:968451. 36081893 10.3389/fnagi.2022.968451 PMC9446483

[27] Sun H, et al. Aftereffect of single transcranial direct and alternating current stimulation on spontaneous home-cage and open-field EEG activities in a mouse model of Alzheimer’s disease. Front Aging Neurosci. 2024;16:1492838. 39737333 10.3389/fnagi.2024.1492838 PMC11683110

[28] Pahor A, Jaušovec N. Making brains run faster: are they becoming smarter? Span J Psychol. 2016;19:E88. 27917748 10.1017/sjp.2016.83

[29] Wang P, et al. The effect of somatosensory alpha transcranial alternating current stimulation on pain empathy is trait empathy and gender dependent. CNS Neurosci Ther. 2021;27(6):687–93. 33739605 10.1111/cns.13631 PMC8111491

[30] Weller S, Derntl B, Plewnia C. Sex matters for the enhancement of cognitive training with transcranial direct current stimulation (tDCS). Biol Sex Differ. 2023;14(1):78. 37919761 10.1186/s13293-023-00561-4 PMC10623760

[31] Toth AJ, et al. The effect of bipolar bihemispheric tDCS on executive function and working memory abilities. Front Psychol. 2023;14:1275878. 38235279 10.3389/fpsyg.2023.1275878 PMC10791995

[32] León JJ, et al. Transcranial direct current stimulation improves risky decision making in women but not in men: a sham-controlled study. Behav Brain Res. 2020;382:112485. 31958518 10.1016/j.bbr.2020.112485

[33] Klink K, et al. The modulation of cognitive performance with transcranial alternating current stimulation: a systematic review of frequency-specific effects. Brain Sci. 2020;10(12):932. 10.3390/brainsci10120932 33276533 10.3390/brainsci10120932 PMC7761592

[34] Booth SJ, et al. The effects of transcranial alternating current stimulation on memory performance in healthy adults: a systematic review. Cortex. 2022;147:112–39. 35032750 10.1016/j.cortex.2021.12.001

[35] Vogeti S, Boetzel C, Herrmann CS. Entrainment and spike-timing dependent plasticity - a review of proposed mechanisms of transcranial alternating current stimulation. Front Syst Neurosci. 2022;16:827353. 35283735 10.3389/fnsys.2022.827353 PMC8909135

[36] Kraft JD, Hampstead BM. A systematic review of tACS effects on cognitive functioning in older adults across the healthy to dementia spectrum. Neuropsychol Rev. 2024;34(4):1165–90. 37882864 10.1007/s11065-023-09621-3 PMC11045666

[37] Zarubin G, et al. Transient amplitude modulation of alpha-band oscillations by short-time intermittent closed-loop tACS. Front Hum Neurosci. 2020;14:366. 33100993 10.3389/fnhum.2020.00366 PMC7500443

[38] He Y, et al. Neurophysiological mechanisms of transcranial alternating current stimulation. Front Neurosci. 2023;17:1091925. 37090788 10.3389/fnins.2023.1091925 PMC10117687

[39] Castilla A, et al. Age and sex impact on visuospatial working memory (VSWM), mental rotation, and cognitive strategies during navigation. Neurosci Res. 2022;183:84–96. 35905778 10.1016/j.neures.2022.07.007

[40] Giofre D, et al. Sex/gender differences in general cognitive abilities: an investigation using the Leiter-3. Cogn Process. 2024;25(4):663–72. 38748044 10.1007/s10339-024-01199-9 PMC11541283

[41] Hu K, et al. Effects of transcranial alternating current stimulation on motor performance and motor learning for healthy individuals: a systematic review and meta-analysis. Front Physiol. 2022;13:1064584. 36467691 10.3389/fphys.2022.1064584 PMC9715745

[42] Rostami M, et al. Determining the corticospinal, intracortical and motor function responses to transcranial alternating current stimulation of the motor cortex in healthy adults: A systematic review and meta-analysis. Brain Res. 2023;148650:p. 10.1016/j.brainres.2023.148650 39491217

[43] Guerra A, et al. Theta-tACS modulates cerebellar-related motor functions and cerebellar-cortical connectivity. Clin Neurophysiol. 2024;158:159–69. 38219405 10.1016/j.clinph.2023.12.129

[44] Guerra A, et al. Driving motor cortex oscillations modulates bradykinesia in Parkinson’s disease. Brain. 2022;145(1):224–36. 34245244 10.1093/brain/awab257

[45] Herzog R, et al. Neuronavigated cerebellar 50 hz tACS: attenuation of stimulation effects by motor sequence learning. Biomedicines. 2023;11(8):2218. 10.3390/biomedicines11082218 37626715 10.3390/biomedicines11082218 PMC10452137

[46] McAleer J, et al. Neuromodulatory effects of transcranial electrical stimulation on emotion regulation in internalizing psychopathologies. Clin Neurophysiol. 2023;145:62–70. 36442377 10.1016/j.clinph.2022.10.015 PMC9772290

[47] Lee TW, Li CR, Tramontano G. Tripod transcranial alternating current stimulation at 5-Hz to alleviate anxiety symptoms: a preliminary report. J Affect Disord. 2024;360:156–62. 38821364 10.1016/j.jad.2024.05.166

[48] Wischnewski M, Alekseichuk I, Opitz A. Neurocognitive, physiological, and biophysical effects of transcranial alternating current stimulation. Trends Cogn Sci. 2023;27(2):189–205. 36543610 10.1016/j.tics.2022.11.013 PMC9852081

[49] Lee TW, Tramontano G. Neural consequences of 5-Hz transcranial alternating current stimulation over right hemisphere: an eLORETA EEG study. Neurosci Lett. 2024;835:137849. 38825146 10.1016/j.neulet.2024.137849

[50] Agboada D, Zhao Z, Wischnewski M. Neuroplastic effects of transcranial alternating current stimulation (tACS): from mechanisms to clinical trials. Front Hum Neurosci. 2025;19:1548478. 40144589 10.3389/fnhum.2025.1548478 PMC11936966

[51] Villa A, et al. Estrogens, neuroinflammation, and neurodegeneration. Endocr Rev. 2016;37(4):372–402. 27196727 10.1210/er.2016-1007 PMC4971309

[52] Vegeto E, et al. The role of sex and sex hormones in neurodegenerative diseases. Endocr Rev. 2020;41(2):273–319. 31544208 10.1210/endrev/bnz005 PMC7156855

[53] Wu L, et al. Long-term gamma transcranial alternating current stimulation improves the memory function of mice with Alzheimer’s disease. Front Aging Neurosci. 2022;14:980636. 36185476 10.3389/fnagi.2022.980636 PMC9520626

[54] Giustiniani A, et al. Functional changes in brain oscillations in dementia: a review. Rev Neurosci. 2022. 10.1515/revneuro-2022-0010. 35724724 10.1515/revneuro-2022-0010

[55] Jiao B, et al. Neural biomarker diagnosis and prediction to mild cognitive impairment and Alzheimer’s disease using EEG technology. Alzheimers Res Ther. 2023;15(1):32. 36765411 10.1186/s13195-023-01181-1 PMC9912534

