# Correction: Sex differences in the effects of 10 Hz and 40 Hz transcranial alternating current stimulation on spatial cognition in mice

**DOI:** 10.1186/s13293-025-00791-8

**Published:** 2025-11-24

**Authors:** Yunbin Zhang, Ping Ren, Zhuangfei Chen, Yu Fu

**Affiliations:** 1 Medical School, Kunming University of Science & Technology, #727 Jing Ming Nan Road, Chenggong County, Kunming, Yunnan, 650500 China; 2 Department of Geriatric Psychiatry, Shenzhen Mental Health Center/Shenzhen Kangning Hospital, Shenzhen, 518020 Guangdong China

**Correction: Biology of Sex Differences (2025) 16:89**

10.1186/s13293-025-00778-5

Following publication of the original article [1], the authors reported some typesetting errors, whereby the figures in the final published article were placed in the incorrect order. The correct order of figures is given below.

Further to this, in Table 2, “P < 0.001###” needs to be bolded. In Table 3, the underlining of certain entries are missing. The incorrect and correct Table 3 is supplied below. The original article [1] has been updated.

**Fig. 1:**
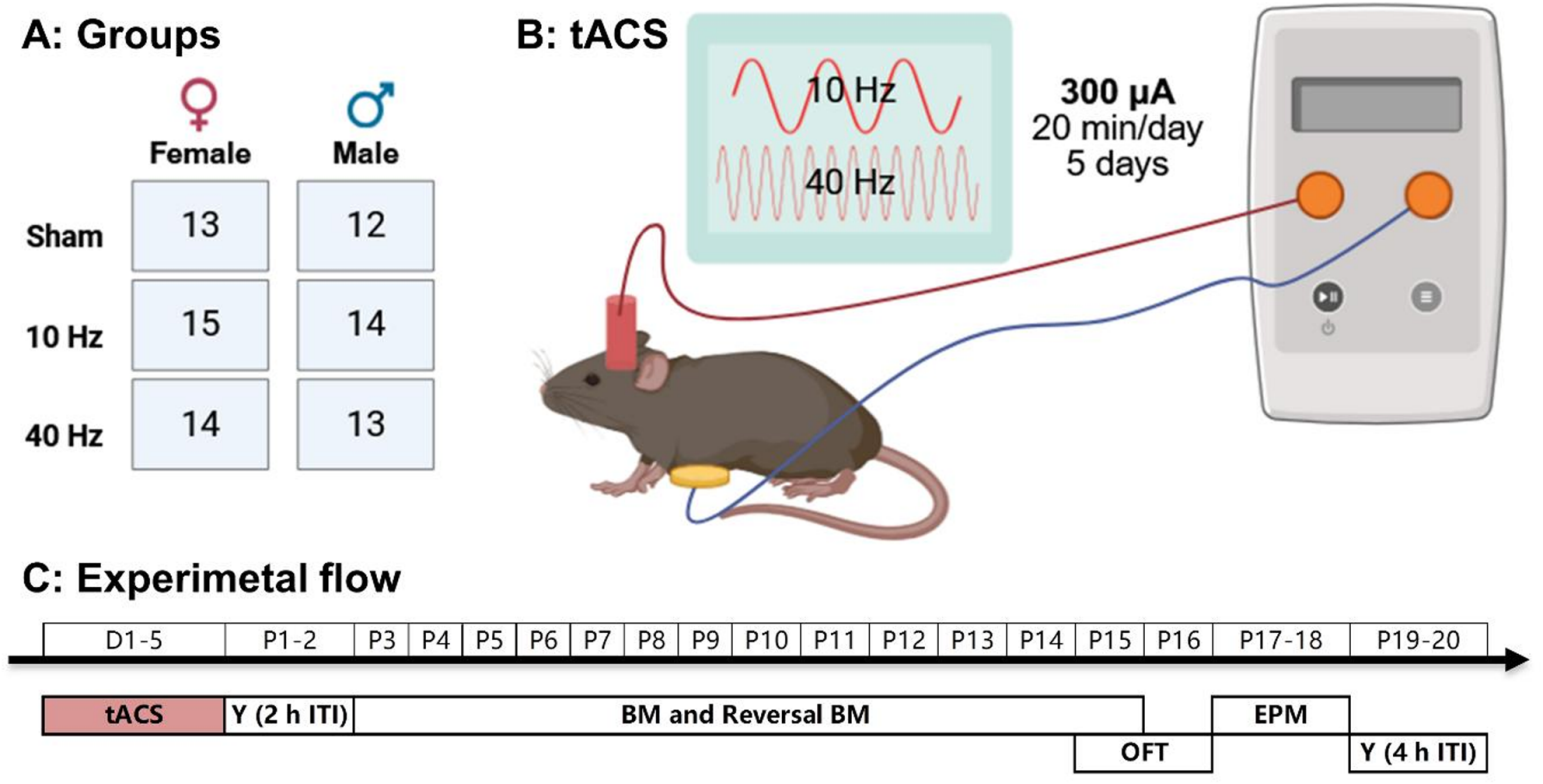
Experimental design. (**A**) Number of mice in each tACS group categorized by sex. (**B**) Each mouse was implanted with a plastic tube on the skull that was filled with saline and used as a tACS electrode. The other electrode was placed on the mouse’s abdomen. (**C**) Experiment flow: D1-D5 and P1-P20 represent the days of tACS and the days after tACS, respectively. Y represents the Y-maze task, BM represents the Barnes maze task, OFT represents the open-field task, and EPM represents the elevated plus maze task. Created in BioRender. Fu, Y. (2025) https://BioRender.com/1gvaicv

**Fig. 2:**
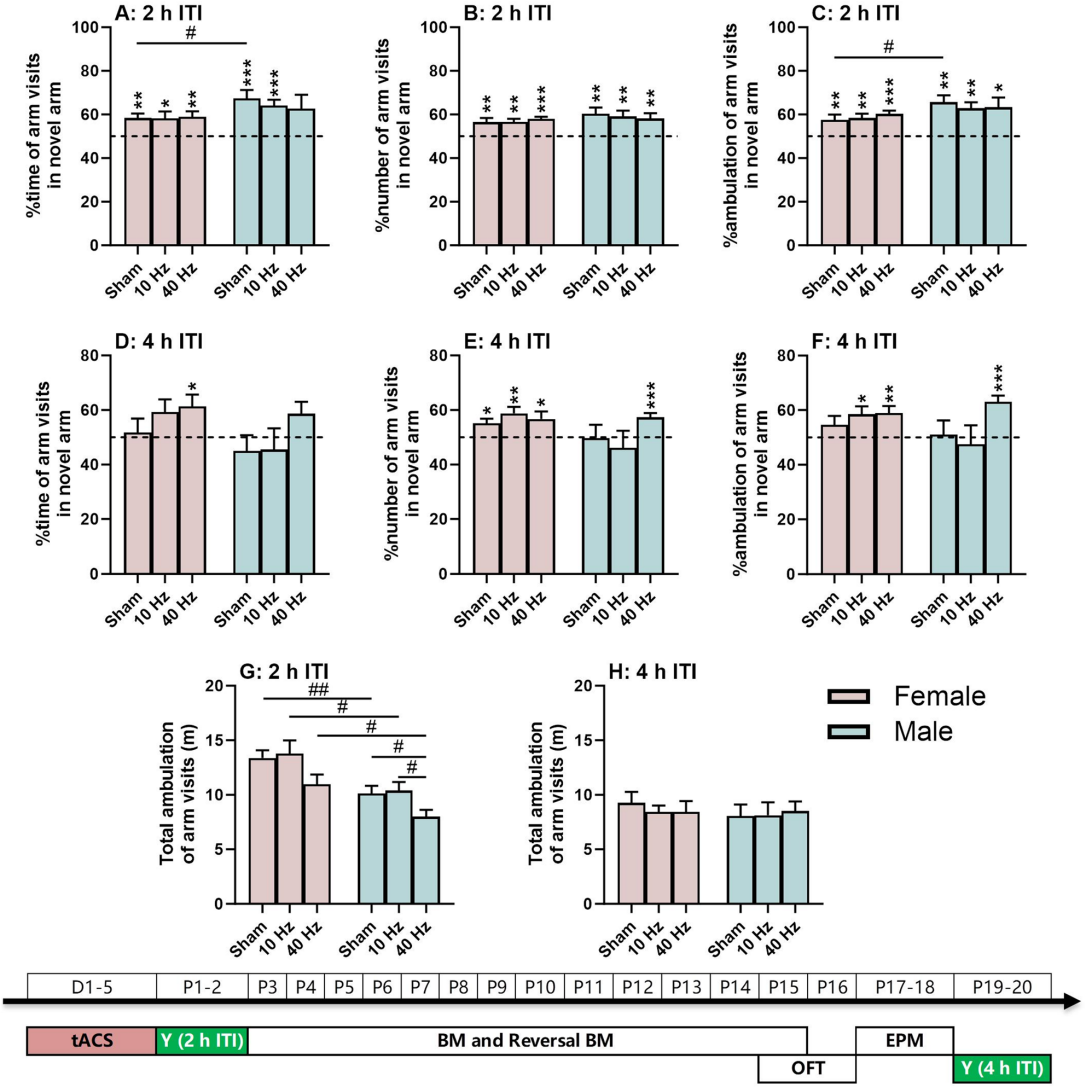
Spatial recognition memory based on the exploratory nature of the animal. (**A**-**C**) Y-maze task with a 2-h inter-trial interval (ITI). (**D**-**F**) Y-maze task with a 4-h ITI. (**G**-**H**) Total ambulation distance in the maze as a locomotor index. The experimental times are shown below the figures in white text on a green background. The (*) symbol indicates significant differences compared to the 50% chance level (dashed line). The (#) symbol indicates post-hoc significant differences between groups. One symbol denotes *P* < 0.05, two symbols denote *P* < 0.01, and three symbols denote *P* < 0.001

**Fig. 3:**
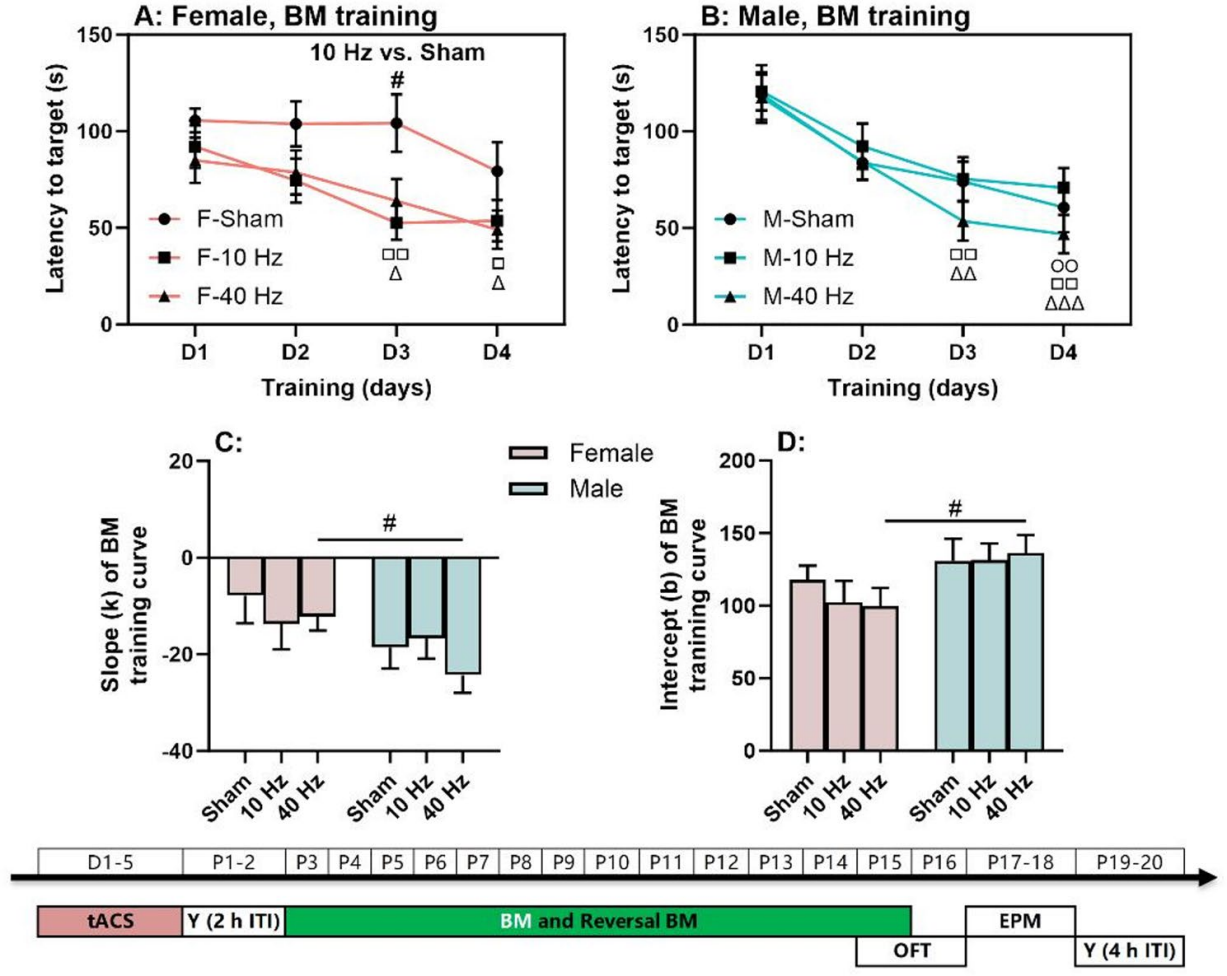
Spatial learning with aversive bright light as punishment. (**A**) Barnes maze (BM) training curves in females. (**B**) BM training curves in males. (**C**-**D**) Slopes and intercepts of training curves in females and males. The experimental times are shown below the figures in white text on a green background. The symbols (○, □, and △) indicate significant differences compared to Day 1 (D1) for the Sham, 10 Hz tACS, and 40 Hz tACS groups, respectively. The (#) symbol indicates significant differences between groups. Post-hoc symbols are shown. One symbol denotes *P* < 0.05, two symbols denote *P* < 0.01, and three symbols denote *P* < 0.001

**Fig. 4:**
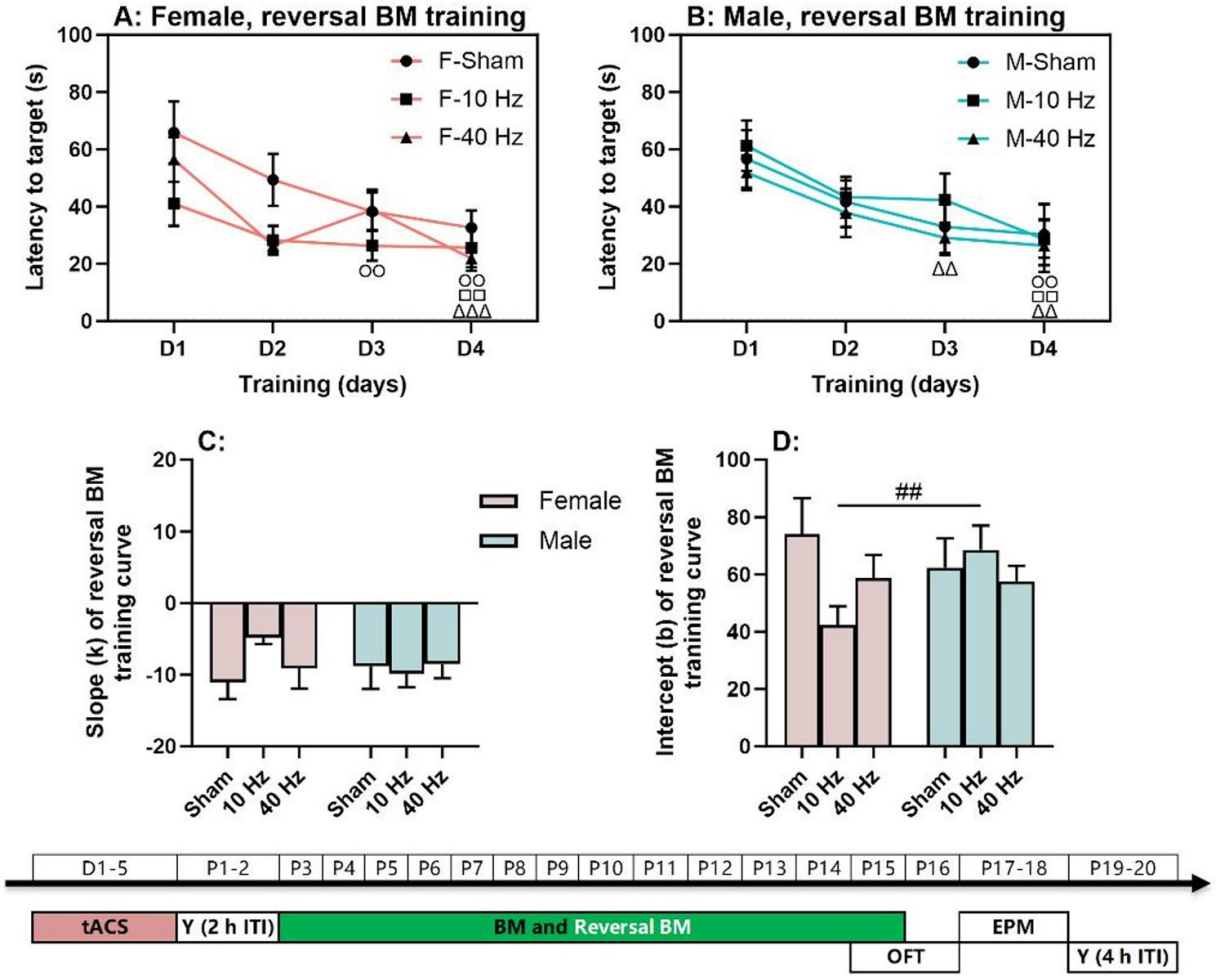
Spatial reversal learning with aversive bright light as punishment. (**A**) Reversal Barnes maze (BM) training curves in females. (**B**) Reversal BM training curves in males. (**C**-**D**) Slopes and intercepts of the reversal training curves in females and males. The experimental times are shown below the figures in white text on a green background. The symbols (○, □, and △) indicate significant differences compared to Day 1 (D1) for the Sham, 10 Hz tACS, and 40 Hz tACS groups, respectively. The (#) symbol indicates significant differences between groups. Post-hoc symbols are shown. One symbol denotes *P* < 0.05, two symbols denote *P* < 0.01, and three symbols denote *P* < 0.001

**Fig. 5:**
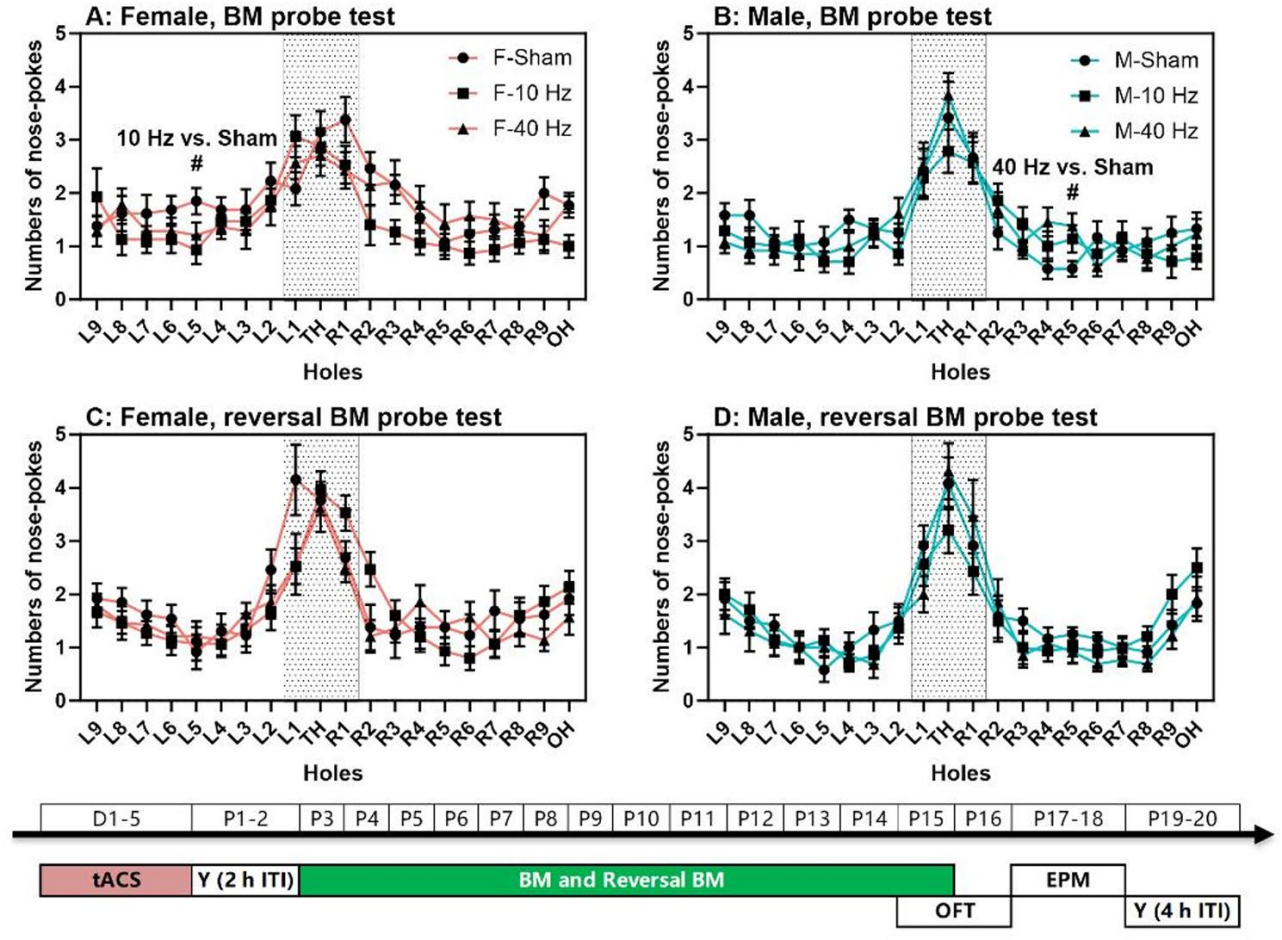
Spatial memory with aversive bright light as punishment. (**A**-**B**) Numbers of nose-pokes to all holes during the Barnes maze (BM) probe test are shown for females and males, respectively. (**C**-**D**) Numbers of nose-pokes to all holes during the reversal BM probe test are shown for females and males, respectively. The experimental times are shown below the figures in white text on a green background. The (#) symbol indicates post-hoc significant differences at *P* < 0.05 between groups

Incorrect Table 3.

**Table 3:** Summary of the effect of tACS on locomotion in females and males

Tasks	Locomotor indices	Sham *n*_f_=13 *n*_m_=12	10 Hz *n*_f_=15 *n*_m_=14	40 Hz *n*_f_=14 *n*_m_=13	(10 Hz-Sham) /Sham*100	(40 Hz-Sham) /Sham*100
**Females**					***−4.589*** ^a^	***−5.226*** ^a^
Y: 2 h ITI	Ambulation (m)	13.375	13.786	10.970	3.070	*−17.984* ^a^
OFT	Ambulation (m)	54.030	47.650	49.605	*−11.808* ^a^	*−8.190* ^a^
EPM	Ambulation (m)	15.551	14.097	14.030	*−9.350* ^a^	*−9.781* ^a^
BM	Speed (mm/s)	91.068	90.781	100.552	*−0.315* ^a^	10.414
Reversal BM	Speed (mm/s)	101.549	96.939	100.949	*−4.540* ^a^	*−0.591* ^a^
**Males**					**−2.370** ^**a**^	**−6.374** ^**a**^
Y: 2 h ITI	Ambulation (m)	10.117	10.396	7.986	2.758	*−21.059* ^a^
OFT	Ambulation (m)	45.530	48.624	48.685	6.796	6.929
EPM	Ambulation (m)	12.407	12.400	11.701	*−0.056* ^a^	*−5.690* ^a^
BM	Speed (mm/s)	85.730	74.350	78.680	*−13.274* ^a^	*−8.223* ^a^
Reversal BM	Speed (mm/s)	90.217	82.935	86.766	*−8.072*	*−3.825*
(Males-Females)/Females*100					**85.731** ^**c**^	**202.537** ^**c**^
Y: 2 h ITI	Ambulation (m)	*−24.363* ^b^	*−24.592* ^b^	*−27.200* ^b^	*0.940* ^c^	*11.641* ^c^
OFT	Ambulation (m)	*−15.732* ^b^	2.044	*−1.855* ^b^	−112.993	−88.211
EPM	Ambulation (m)	*−20.217* ^b^	*−12.038* ^b^	*−16.600* ^b^	−40.457	−17.892
BM	Speed (mm/s)	*−5.862* ^b^	*−18.100* ^b^	*−21.752* ^b^	*208.785* ^c^	*271.095* ^c^
Reversal BM	Speed (mm/s)	*−11.159* ^b^	*−14.446* ^b^	*−14.050* ^b^	*29.456* ^c^	*25.903* ^c^

n_f_ and n_m_: Number of females and males in each tACS group, respectively

Bold values: The averaged changes calculated from the 5 normalized values below the value

^a^**:**Underlined negative values indicate decreased locomotion in the tACS frequency group relative to the sham group

^b^**: **Underlined negative values indicate decreased locomotion in the males relative to females

^c^**: **Underlined positive values indicate decreased locomotion in the tACS frequency group relative to the sham group

Correct Table 3.

**Table 3** Summary of the effect of tACS on locomotion in females and males.

**Table:** 

Tasks	Locomotor indices	Sham *n*_f_=13 *n*_m_=12	10 Hz *n*_f_=15 *n*_m_=14	40 Hz *n*_f_=14 *n*_m_=13	(10 Hz-Sham) /Sham*100	(40 Hz-Sham) /Sham*100
**Females**					***−4.589*** ^a^	***−5.226*** ^a^
Y: 2 h ITI	Ambulation (m)	13.375	13.786	10.970	3.070	*−17.984* ^a^
OFT	Ambulation (m)	54.030	47.650	49.605	*−11.808* ^a^	*−8.190* ^a^
EPM	Ambulation (m)	15.551	14.097	14.030	*−9.350* ^a^	*−9.781* ^a^
BM	Speed (mm/s)	91.068	90.781	100.552	*−0.315* ^a^	10.414
Reversal BM	Speed (mm/s)	101.549	96.939	100.949	*−4.540* ^a^	*−0.591* ^a^
**Males**					**−2.370** ^**a**^	**−6.374** ^**a**^
Y: 2 h ITI	Ambulation (m)	10.117	10.396	7.986	2.758	*−21.059* ^a^
OFT	Ambulation (m)	45.530	48.624	48.685	6.796	6.929
EPM	Ambulation (m)	12.407	12.400	11.701	*−0.056* ^a^	*−5.690* ^a^
BM	Speed (mm/s)	85.730	74.350	78.680	*−13.274* ^a^	*−8.223* ^a^
Reversal BM	Speed (mm/s)	90.217	82.935	86.766	*−8.072*	*−3.825*
(Males-Females)/Females*100					**85.731** ^**c**^	**202.537** ^**c**^
Y: 2 h ITI	Ambulation (m)	*−24.363* ^b^	*−24.592* ^b^	*−27.200* ^b^	*0.940* ^c^	*11.641* ^c^
OFT	Ambulation (m)	*−15.732* ^b^	2.044	*−1.855* ^b^	−112.993	−88.211
EPM	Ambulation (m)	*−20.217* ^b^	*−12.038* ^b^	*−16.600* ^b^	−40.457	−17.892
BM	Speed (mm/s)	*−5.862* ^b^	*−18.100* ^b^	*−21.752* ^b^	*208.785* ^c^	*271.095* ^c^
Reversal BM	Speed (mm/s)	*−11.159* ^b^	*−14.446* ^b^	*−14.050* ^b^	*29.456* ^c^	*25.903* ^c^

n_f_ and n_m_: Number of females and males in each tACS group, respectively

Bold values: The averaged changes calculated from the 5 normalized values below the value

^a^:Underlined negative values indicate decreased locomotion in the tACS frequency group relative to the sham group

^b^: Underlined negative values indicate decreased locomotion in the males relative to females

^c^: Underlined positive values indicate decreased locomotion in the tACS frequency group relative to the sham group
